# Ethnic and Gender Variations in Ischemic Stroke Patterns among Arab Populations in Northern Israel: A Preliminary Exploration towards Culturally Aware Personalized Stroke Care

**DOI:** 10.3390/jpm14050526

**Published:** 2024-05-15

**Authors:** Chen Hanna Ryder, Carmit Gal, Gili Barkay, Shani Raveh Amsalem, Ziv Sarusi, Radi Shahien, Samih Badarny

**Affiliations:** 1 Brain & Behavior Research Institute, Western Galilee Academic College, Akko 2412101, Israel; 2 The Max Stern Yezreel Valley College, Emek Yezreel 1930600, Israel; 3 Shaanan College, Haifa 26109, Israel; 4 Department of Neurology, Ziv Medical Center, Safed 1311001, Israel; 5 Azrieli Faculty of Medicine, Bar Ilan University, Safed 1311502, Israel; 6 Department of Neurology, Galilee Medical Center, Nahariya 2210001, Israel

**Keywords:** stroke, ethnic, gender, Arab, personalized care, risk factors, epidemiological, stroke severity, stroke type, recurrent stroke, Israel

## Abstract

The Galilee region of Israel boasts a rich ethnic diversity within its Arab population, encompassing distinct Muslim, Christian, Druze, and Bedouin communities. This preliminary exploratory study uniquely examined potential ethnic and gender differences in ischemic stroke characteristics across these Arab subgroups, which are seldom investigated separately in Israel and are typically studied as a homogeneous “Arab” sector, despite significant variations in their ethnicity, culture, customs, and genetics. The current study aimed to comparatively evaluate stroke characteristics, including recurrence rates, severity, and subtypes, within and across these distinct ethnic groups and between genders. When examining the differences in stroke characteristics between ethnic groups, notable findings emerged. The Bedouin population exhibited significantly higher rates of recurrent strokes than Muslims (M = 0.55, SD = 0.85 vs. M = 0.25, SD = 0.56; *p* < 0.05). Large vessel strokes were significantly more prevalent among Christians (30%) than Druze (9.9%; *p* < 0.05). Regarding gender differences within each ethnic group, several disparities were observed. Druze women were six times more likely to experience moderate to severe strokes than their male counterparts (*p* < 0.05). Interestingly, Druze women also exhibited a higher representation of cardio-embolic stroke (19.8%) compared with Druze men (4.6%; *p* < 0.001). These findings on the heterogeneity in stroke characteristics across Arab ethnic subgroups and by gender underscore the need to reconsider the approach that views all ethnic groups comprising the Arab sector in Israel as a homogeneous population; instead, they should be investigated as distinct communities with unique stroke profiles, requiring tailored culturally aware community-based prevention programs and personalized therapeutic models. The identified patterns may guide future research to develop refined, individualized, and preventive treatment approaches targeting the distinct risk factors, healthcare contexts, and prevention needs of these diverse Arab populations.

## 1. Introduction

The present study aimed to conduct a comparative assessment of ischemic stroke characteristics, including recurrence rates, severity, and subtypes, across distinct Arab ethnic communities and between genders within the Galilee region of Israel. These minority populations, comprising Muslims, Christians, Druze, and Bedouins, are often aggregated and considered a homogeneous “Arab” sector in research, despite exhibiting significant variations in ethnicity, culture, customs, lifestyle, and genetics. As these unique subgroups predominantly reside in the Galilee region, a northern periphery area remote from Israel’s main population centers, they have rarely been the focus of dedicated investigations, providing this preliminary exploratory study with a rare opportunity to examine their specific stroke profiles.

This research offered a unique window into these diverse Arab ethnic communities, enabling a comparative analysis of the distinct subpopulations that constitute the Arab societal fabric of Israel. This study was conducted at the two sole neurological departments serving the Galilee region—the Galilee Medical Center and the Ziv Medical Center—where these small unique ethnic groups are concentrated, allowing for a comprehensive investigation of the differences in stroke severity, subtypes, and recurrence rates across these subgroups, while also accounting for gender-based distinctions.

Ethnic differences in the context of stroke have emerged as a subject of considerable interest. Currently, extensive research has been conducted on the discrepancies between African American and Caucasian populations in the United States regarding stroke, encompassing aspects such as risk factors, incidence, and mortality [1,2,3]. These investigations have revealed not only a higher incidence of stroke among Afro-Americans compared with Caucasians but also a discernible upward trend, particularly among young adults [4]. Furthermore, studies have highlighted an elevated prevalence of hypertension and diabetes mellitus throughout the lifespan of black patients in contrast to their white counterparts [5].

With respect to gender, a study comparing women and men found that white women were disproportionately represented by the highest stroke severity, whereas white men were represented by the lowest severity [6,7,8]. This finding is consistent with the observation that women tend to have more severe strokes but better survival rates than men [6]. The differences in stroke severity between men and women may be attributed to sex-specific risk factors and biological determinants [9]. For instance, women have been found to have higher levels of procoagulant factors, while men exhibit increased levels of coagulation inhibitors, which may contribute to variations in stroke severity [10]. Furthermore, the neuroprotective effects of estrogen have been suggested as a potential explanation for sex differences in stroke severity, as evidenced by increased P450 aromatase levels in post-menopausal women after acute ischemic stroke [11]. The variability observed among ethnic and gender groups in terms of risk factors has been ascribed to an intricate interplay of environmental and genetic factors [9,12]. In the State of Israel, a declining trend in stroke occurrences has been identified, with a more pronounced decline among Jews compared with Arabs [13,14]. Limited studies exploring risk factors, major etiologies, and diagnostic methods among different ethnic groups in Israel (Jews and Arabs) have revealed variations in the prevalence of these factors and outcomes of diagnostic tests [13,15,16]. Notably, differences were observed in the prevalence of risk factors, with higher rates of high blood pressure and diabetes among Arabs compared with Jews [13]. Much of the research conducted in the State of Israel has focused on exploring differences between the two primary ethnic groups—Jews and Arabs [15,17,18]—or within specific subgroups of these populations (e.g., [19]). An Israeli study [19] found a higher prevalence of risk factors such as smoking, dyslipidemia, diabetes mellitus, and hypertension among Bedouin Arabs, underscoring the importance of addressing specific risk factors within distinct ethnic groups. Furthermore, differences in stroke risk factors and outcomes have been observed between Israeli Arabs and Jewish Israelis. Another Israeli study [20] pointed out low to moderate levels of stroke knowledge among Arab Muslim Israelis, indicating a potential gap in awareness and preventive practices. It was also reported [21] that Israeli Arabs experience strokes at younger ages and with more severe outcomes compared with Israeli Jews, attributing this difference to higher risk factor prevalence among Arab populations.

However, such studies often overlook the nuances of diversity within the Arab minority, which comprises various distinct ethnic groups such as Druze, Bedouin, and Christian Arabs. Treating these groups as a homogeneous entity labeled “Arabs in Israel” may potentially impede the development of intervention plans and specific strategies tailored to the unique characteristics and motivations of each subgroup. This preliminary exploratory study therefore aims to characterize the unique ischemic stroke attributes in each of the prevalent Arab subpopulations.

By elucidating these distinctions, the findings may help guide future research to advance preventive medicine and inform the development of personalized medicine models specific to each group rather than treating them as a monolithic population. Through this novel comparative analysis, this study highlights the importance of recognizing the heterogeneity within the Arab population and provides crucial insights to steer subsequent investigations towards designing more customized and effective stroke prevention and management strategies for these diverse ethnic communities.

## 2. Method

### 2.1. Study Design

This retrospective study was conducted at the Brain and Behavior Research Institute, Western Galilee Academic College in 2023. This investigation employed a quantitative approach, involving the structured collection and statistical analysis of numerical data extracted from patient medical records. Variables examined included stroke severity scores, recurrence rates, and stroke subtypes. Comparative analyses were performed to identify significant differences in these measures between the various Arab ethnic groups and between genders within each group.

### 2.2. Data Source and Participants

This study utilized an existing database comprising the medical records of patients who were discharged from the Neurology Departments of the Galilee Medical Center and Ziv Medical Center with a primary diagnosis of ischemic stroke during the 2015–2018 period. Patients who were admitted to these two neurology departments, which are the sole providers of care for patients with ischemic stroke in the entire Galilee region, were included in the analysis.

The inclusion criteria were as follows:

- Patients admitted to the Neurology Departments of the Galilee Medical Center or Ziv Medical Center, which are the sole providers of neurological care in the Galilee region, during the years 2015–2018.
- Patients with a primary diagnosis of ischemic stroke as the reason for admission.
- Patients diagnosed specifically with ischemic stroke, rather than hemorrhagic stroke.

Exclusion criteria were as follows:

- Patients admitted with a primary diagnosis other than ischemic stroke.
- Patients diagnosed with hemorrhagic stroke.

The use of these pre-pandemic data from 2015 to 2018 was advantageous, as it circumvented potential biases and inconsistencies introduced by the disruptions to routine medical care and changes in health-seeking behaviors observed during the COVID-19 era starting in 2020.

### 2.3. Data Collection

This retrospective study utilized an existing database comprising medical records of Arab patients diagnosed with stroke and discharged from the Neurology Departments of the Galilee Medical Center and Ziv Medical Center between 2015 and 2018. The database contained comprehensive information on 374 patients, including details on their ethnic subgroup (Muslim, Christian, Druze, Bedouin), stroke characteristics (type, severity, recurrence), demographics, and clinical data. As the sole neurological facilities for the Arab population in northern Israel, these two centers maintain detailed records for all stroke patients receiving care, enabling a complete regional analysis across the diverse Arab subgroups.

Approval for this study was obtained from the local Helsinki ethics committees at the Galilee Medical Center and the Ziv Medical Center. Given this study’s reliance on pre-existing data, the necessity of using informed consent forms was considered unwarranted. The main variables examined were gender, age, number of recurring strokes, stroke type, ethnic group, and stroke severity. Recurring strokes were measured by reviewing patients’ medical histories documented in their records, which consolidated all previous hospitalizations and stroke diagnoses. Stroke types were differentiated based on the medical diagnosis found in the patient’s record following brain imaging with CT, CT angiography, and MRI.

### 2.4. Statistical Analysis

All statistical analyses were performed using SPSS version 28.0 (IBM Corp., Armonk, NY, USA). Descriptive statistics were used to characterize the study population. Continuous variables were expressed as mean ± standard deviation (SD), while categorical variables were presented as frequencies and percentages. To compare the different ethnic groups on the continuous variable of the number of recurring strokes, a one-way analysis of variance (ANOVA) was conducted. The combined effect of gender and ethnic group on the number of recurring strokes was assessed using a two-way ANOVA. Chi-square tests were employed to examine differences in stroke severity between ethnic groups, as well as to investigate differences in stroke severity between genders within each ethnic group. Additionally, Chi-square tests were used to analyze differences in stroke type frequencies among ethnic groups and between genders within each group. In cases where expected cell counts were less than 5, Fisher’s exact test was applied. For all analyses, a *p*-value of less than 0.05 was considered statistically significant.

## 3. Results

### 3.1. Characteristics of the Population

The ethnic breakdown within the cohort revealed that 232 individuals (62%) identified as Druze, 82 (21.9%) as Muslims, 40 (10.7%) as Bedouins, and the remaining 20 (5.3%) as Christians. The mean age for Muslims was 66.15 (SD ± 13.78), for Christians 66.60 (SD ± 15.05), for 154 Druze 66.07 (SD ± 13.33), and for Bedouins 65 (SD ± 12.76). No significant difference in participant ages was observed between the research groups (*p* > 0.05). It is important to note that stroke incidence at this age may be influenced by various genetic, lifestyle, sociocultural, and environmental factors, including sex differences, traditional risk factors, socioeconomic status, access to healthcare, and genetic predispositions within specific ethnic groups [13,19].

### 3.2. Differences in the Number of Recurring Strokes

Initially, differences in the number of recurring strokes were assessed among various ethnic populations using a one-way ANOVA test with a single independent variable (ethnic group). The test results are presented in Table 1.

The test results reveal significant differences in the number of recurring strokes among the various groups (*p* < 0.05). Post hoc comparisons utilizing the Bonferroni correction indicated that Bedouins exhibited a significantly higher average number of recurring strokes (M = 0.55, SD = 0.85) compared with Muslims (M = 0.25, SD = 0.56). Moreover, a notable trend (^ *p* = 0.07) suggested that Bedouins also had a significantly higher number of recurring strokes compared with the Druze population (M = 0.29, SD = 0.62). Subsequently, the combined effect of gender and ethnic group (excluding the Christian group, which constituted a minority in this sample) on the number of recurring strokes was examined (ethnic group × gender) by using a two-way ANOVA test. The results yield a significant interaction effect (*p* < 0.05).

### 3.3. Interaction Effect between Gender and Ethnic Group on the Number of Recurrent Strokes

To evaluate the interaction effect concerning the ethnic group, two-way ANOVA analyses were conducted separately for each gender group. Due to the small sample size in the Christian group, individuals from this group were excluded from this analysis.

**Figure 1:**
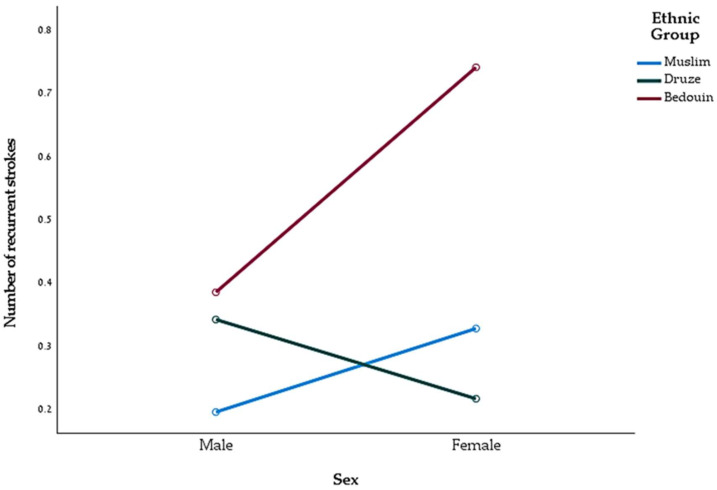
Interaction effect between gender and ethnic group on the number of recurrent strokes.

The results are presented in Figure 1.

Figure 1 illustrates the interaction effect between gender and ethnic group on the number of recurrent strokes. The interaction analysis among males did not reveal a significant difference in the number of recurring strokes among the ethnic groups (*p* > 0.05). However, among females, a significant difference was found among the ethnic groups in the number of recurring brain strokes (*p* < 0.01). A post hoc test of Bonferroni corrected analysis revealed a significant difference between Druze and Bedouin women (*p* < 0.05). Bedouin women exhibited a higher average number of recurring strokes (M = 0.74, SD = 0.93) compared with Druze women (M = 0.21, SD = 0.47). Additionally, a trend was observed indicating a difference between Bedouin and Muslim women in the number of recurring brain strokes (*p* = 0.059). According to this trend, Muslim women exhibited a lower average number of recurring strokes (M = 0.32, SD = 0.684) compared with Bedouin women.

### 3.4. Differences in the Severity of Stroke According to the NIHSS (National Institutes of Health Stroke Scale) Measure

To compare the severity of strokes among different ethnic groups, a Chi-square test was conducted, and Fisher’s exact test was applied to each category of stroke severity. The results of the tests are presented in a Table 2.

### 3.5. Differences in the Severity of Stroke between Genders within Each Examined Ethnic Group

**Figure 2:**
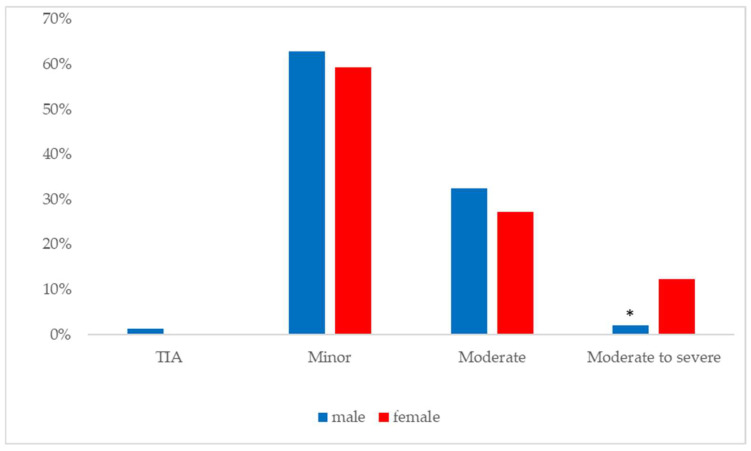
Gender differences in stroke severity among the Druze population. * Significance *p* < 0.05.

To examine the existence of differences in the severity of strokes between women and men within each of the studied ethnic groups, Chi-square tests were conducted, and Fisher’s exact test was applied to each category of stroke severity. The results of the tests are presented in Table 3:

Figure 2 illustrates the gender differences in the prevalence of moderate to severe stroke among the Druze population. Significance results revealed that, while 12.3% of all stroke cases among Druze women were classified as moderate to severe, only 2% of cases among Druze men fell into this severity category. This difference represents a six-fold higher risk for Druze women to experience a moderate to severe stroke relative to Druze men, a statistically significant discrepancy (*p* < 0.05, Fisher’s exact test).

### 3.6. Differences in Frequency of Stroke Type among Each Ethnic Group

The test results regarding the existence of differences in the frequency of stroke type among the researched ethnic groups (without gender division) are presented in Table 4.

The test results examining the presence of differences among the ethnic subgroups in the type of stroke indicated that the frequency of large vessel-type stroke was significantly higher in the Christian population (30%) compared with the Druze population (9.9%) (*p* < 0.05, FET). Additionally, a Chi-square test revealed a trend suggesting a higher frequency of small vessel-type stroke among Bedouins (67.5%) compared with Druze individuals (51%) (*p* = 0.058).

### 3.7. Differences in Frequency of Stroke Type between Male and Female among Each Ethnic Group

Results of examining differences in the type of stroke between women and men within each ethnic group are presented in Table 5.

## 4. Discussion

In this study, a thorough comparison was undertaken concerning the number of recurrent strokes, the severity of strokes, and the types of strokes among different ethnic groups and between women and men within the Arab population seeking treatment at the Galilee Medical Center and the Ziv Medical Center. This investigation offers insight into ethnic variations in ischemic stroke recurrence, severity, subtype, and sex predilection within the heterogeneous Arab population residing in northern Israel.

Existing studies conducted in Israel primarily focus on comparing two main ethnic groups—Arabs and Jews—often addressing the Arab society as a homogenous group and not referring to the division as different ethnic subgroups, namely Druze, Muslim, Christian, and Bedouin populations. Furthermore, from a medical perspective, there is a notable lack of comprehensive examination in the available literature regarding differences in stroke patterns between women and men from various ethnic groups. The present study serves as a preliminary exploration, providing insight into the distinctions among subgroups within the Arab community in the north of Israel while taking into account gender differences in the manifestation of strokes.

### 4.1. Recurrent Number of Strokes

Analysis of the research results revealed that, within the Bedouin population, there was a higher prevalence of recurrent number of strokes compared with Muslims and Druze. Additionally, in the comparison among women, it was found that Bedouin women experienced a higher frequency of recurrent strokes compared with Druze and Muslim women. However, no differences were found in the number of recurrent strokes among men from different ethnic groups.

Studies have demonstrated that secondary prevention using antiaggregant medications can significantly reduce the risk of recurrent ischemic stroke [22,23]. However, research indicates Bedouin women show greater preference for alternative healthcare services such as homeopathy and herbal remedies compared with conventional medicine for managing chronic conditions [24]. This suggests that Bedouin women may underutilize standard therapies like antihypertensives, antiplatelets, and lipid-lowering agents generally recommended for secondary stroke prevention. If accurate, this pattern among Bedouin women could partially explain the higher rates of recurrent strokes seen in this subgroup compared with Druze and Muslim populations.

### 4.2. Stroke Severity

These research findings indicated no differences in stroke severity between ethnic groups. However, when differences between genders within each ethnic group were examined regarding the frequency of moderate to severe strokes, disparities were found only among the Druze population. It was found that Druze women had a six-fold higher probability of experiencing moderate to severe strokes compared with Druze men. Various studies have found a significant association between different health problems—including cardio-vascular issues—and unemployment; however, a causal relationship between the two has not been established [25,26]. It can be assumed that unemployment is associated with a sedentary lifestyle and less physical activity, while the cultural/traditional role of the mature Druze woman involves extensive daily activity around household chores. Druze women may be less knowledgeable about the clinical symptoms of stroke, tending to seek medical attention later at the emergency department, consequently with a higher severity of stroke.

### 4.3. Type of Stroke

The findings revealed significant ethnic differences in ischemic stroke subtypes. The Christian population exhibited a significantly higher frequency of large vessel-type strokes compared with the Druze population. Additionally, a trend emerged suggesting an increased prevalence of small vessel-type strokes among Bedouins relative to Druze individuals. These differences in stroke subtypes across ethnic groups, such as the elevated incidence of large vessel strokes in Christians compared with Druze and the increased occurrence of small vessel strokes in Bedouins compared with Druze, may stem from an intricate interplay of genetic, lifestyle, and socioeconomic factors.

Differences in the type of stroke can provide valuable insights into the distribution of risk factors for stroke. Indeed, for each subtype of stroke, there exists a distinct risk factor profile [27]. An early German study [28] examined the prevalence of risk factors for stroke across different subtypes and found differences in the frequency of childbirth, smoking, and heart diseases. The risk factor profile was distributed as follows: childbirth was present in 72% of the small vessel disease group, 57% in the cardio-embolic (CE) group, and 52% in the large vessel disease group. Additionally, 25% of the large vessel disease group were smokers, compared with only 18% and 8% in the small vessel disease and CE groups, respectively. Furthermore, 81% of the CE group had some form of heart disease, while only 45% in the large vessel disease group and 33% in the small vessel disease group did.

In examining gender differences within each ethnic population, a striking observation emerged. Druze women were more represented by cardio-embolic strokes (19.8%) compared with Druze men (4.6%). Studies [29,30] have highlighted that women with atrial fibrillation (AF) may be at a higher risk of thrombo-embolic events and systemic embolism, indicating a propensity towards cardio-embolic strokes. These findings may be explained by the higher prevalence of heart disease, with an emphasis on atrial fibrillation, among women in general and, in particular, Druze women compared with men. It is also possible that women in the general Arab population, and Druze women in particular, have lower compliance with the treatment of underlying heart conditions as suggested in an earlier study [31], leading to a higher representation of cardio-embolic strokes.

To summarize, the literature supports the notion of gender differences in stroke subtypes, with women often exhibiting a higher prevalence of cardio-embolic strokes. The observation of Druze women being more represented by cardio-embolic strokes compared with Druze men aligns with existing research on sex-specific differences in stroke characteristics, emphasizing the need for further investigation into these disparities within specific ethnic populations. Addressing these disparities and their underlying causes holds profound implications for tailoring preventive strategies and optimizing clinical management approaches.

### 4.4. Study Limitations

The present study’s findings regarding ethnic and gender differences in ischemic stroke subtypes should be interpreted with consideration of several limitations. First, the observed differences in stroke type and severity across ethnic and gender groups may be influenced by the sample size and the retrospective design of the study. Larger more diverse samples from multiple regions, along with a prospective longitudinal study design following participants over an extended period, could provide a more comprehensive understanding of the potential genetic, lifestyle, and socioeconomic factors contributing to stroke variations. Therefore, the generalizability of these findings may be limited, and caution should be exercised when extrapolating these results to broader populations or over extended periods beyond the 2015–2018 window examined here [32]. Another limitation of this study is the paucity of data regarding accessibility to medical care and specific socioeconomic status (SES) information across the various ethnic groups examined. The retrospective nature of this study, based on an examination of patient files, limited our ability to gather specific data on participants’ socioeconomic status. This lack of detailed SES information for individual participants hinders the exploration of potential associations between socioeconomic factors and stroke outcomes, warranting further investigation in future studies. SES and economic factors are known to exert a profound influence on the ability to receive timely and adequate medical attention, which may consequently impact stroke outcomes [33]. Despite the generally lower socioeconomic conditions experienced by all minority groups in the Galilee region, variations in healthcare accessibility within and between these communities cannot be disregarded. Differences in access to specialized stroke centers or the ability to promptly seek medical evaluation and treatment may potentially contribute to the observed ethnic and gender-based differences in stroke severity and prognosis.

## 5. Conclusions and Future Directions

This preliminary study thoroughly examined the intricate interplay between ethnic origin, recurrent strokes, type, and severity of stroke, offering a nuanced comparison between men and women within each subgroup. The comprehensive participant cohort of 373 individuals constituted the entirety of the treated population from the Arab population seeking care at both the Galilee Medical Center and the Ziv Medical Center, the two northernmost hospitals in Israel, following strokes between 2015 and 2018. This investigation scrutinized differences among subgroups within the Arab population, encompassing Druze, Arab Muslims, Bedouins, and Arab Christians, while also delving into gender-specific variations. The outcomes of this study shed light on notable distinctions between these subgroups concerning the stipulated stroke criteria. These differences suggest a divergent distribution of risk factors within the diverse ethnic groups and between the genders.

Modern Western medicine is oriented toward preventive care, striving to establish models for personalized and precisely defined healthcare. However, in the absence of information on specific risk factors characterizing each ethnic and gender group, the medical system may overlook crucial aspects essential for preventing and mitigating strokes in a targeted manner for specific ethnic or gender groups. At the practical level, the medical team bears the responsibility of adopting a perspective that enables them to recognize the ethnic, cultural, and gender components inherent in each patient. This approach involves considering the medical implications that may be pertinent to these components. By doing so, we can advance further into the realm of personalized medicine, treating individuals holistically and acknowledging the ethnic, cultural, and gender-related elements that are pertinent to medical interventions. It is vital for us as a medical team to cultivate a comprehensive understanding that enables us to perceive each patient as a distinct individual, taking into account their ethnic, cultural, and gender-specific components. This targeted approach holds the potential to enhance the effectiveness of medical interventions, resulting in improved patient outcomes and overall healthcare efficiency.

## Figures and Tables

**Table 1:** Differences in the number of recurring brain strokes among the researched ethnic subgroups.

Number of Recurrent Strokes	Druze (n = 232)		Muslim (n = 82)		Bedouin (n = 40)		Christian (n = 20)		*p*
	M	SD	M	SD	M	SD	M	SD	
	^ 0.29 ^a^	0.62	0.25 ^a^	0.56	0.55 ^b^	0.85	0.45 ^ab^	0.68	* <0.05

M—mean, SD—standard deviation. The letters indicate significantly higher (^b^), lower (^ab^), or lowest (^a^). * Significant *p* < 0.05, ^ trend 0.05 < *p* < 0.071.

**Table 2:** Differences in the severity of stroke according to the NIHSS questionnaire among the studied ethnic groups.

Stroke Severity	Minority Communities								*p*-Value from Chi^2^ Test
	Druze (N = 232)		Muslim (N = 81)		Bedouin (N = 40)		Christians (N = 20)		
	N	%	N	%	N	%	N	%	
TIA	2	0.9%	1	1.2%	0	0%	0	0%	0.96 (FET ^1^)
Minor	143	61.6%	47	58%	24	6%	13	65%	0.64
Moderate	71	30.6%	28	34.5%	11	27.5%	6	30%	0.77
Moderate to Severe	13	5.6%	1	1.23%	2	5%	2	10%	0.92 (FET ^1^)

^1^ FET—Fisher exact test was performed in case one of the cells contained less than 5 observations. No significant changes were found in stroke severity between the different ethnic groups.

**Table 3:** Differences in the severity of stroke between genders within each examined ethnic group.

Minority Communities	Sex									*p*-Value from Chi^2^ Test
		TIA		Minor		Moderate		Moderate to Severe		
		N	%	N	%	N	%	N	%	
Druze (n = 232)	Male (n = 151)	2	1.30%	95	62.90%	49	32.5%	3	2% ^a^	<0.05 *
	Female (n = 81)	0	0%	48	59.30%	22	27.2%	10	12.3% ^b^	**(FET ^1^)**
Muslim (n = 81)	Male (n = 47)	0	0%	28	59.60%	16	34%	0	0%	**0.93**
	Female (n = 34)	1	2.90%	19	55.90%	12	35.30%	1	2.90%	
Bedouin (n = 40)	Male (n = 21)	0	0%	14	66.70%	5	23.80%	1	4.80%	**0.88**
	Female (n = 19)	0	0%	10	52.60%	6	31.60%	1	5.30%	
Christians (n = 20)	Male (n = 13)	0	0%	8	61.50%	4	30.80%	1	7.70%	**0.73**
	Female (n = 7)	0	0%	5	71.40%	2	28.60%	1	5.30%	**(FET ^1^)**

* Significance *p* < 0.05, =The letters indicate significantly higher (^b^), or lowest (^a^). ^1^ FET—Fisher exact test was performed in case one of the cells contained less than 5 observations. In order to compare males and females regarding differences in the severity of stroke within each ethnic population, Fisher’s exact test was conducted. It was found that Druze women had a 6-fold higher likelihood of experiencing moderate to severe stroke compared with Druze men (<0.05, FET).

**Table 4:** Differences in frequency of stroke type among each ethnic group.

Stroke Subtype	Minority Communities								*p*-Value from Chi^2^ Test
	Druze		Muslim		Bedouin		Christians		
	(N = 232)		(N = 82)		(N = 40)		(N = 20)		
	N	%	N	%	N	%	N	%	
Large vessel	23	9.9% ^a^	10	12.2% ^ab^	4	10% ^a^	6	30% ^b^	*p* < 0.05 *
									(FET) ^1^
Cardio-Embolic	23	9.90%	5	6.10%	1	2.50%	2	10%	0.87
Small vessel	119	51.3% ^a^	48	58.5% ^ab^	27	67.5% ^b^	9	45% ^a^	0.058 ^
TIA\Uncertain	48	20.70%	12	14.60%	4	10%	2	10%	0.67
Intracerebral Hemorrhage	13	5.60%	2	2.40%	2	5%	1	5%	0.75
									(FET) ^1^

* Significance *p* < 0.05, ^ trend 0.05 < *p* < 0.071. The letters indicate significantly higher (^b^), lower (^ab^), or lowest (^a^). ^1^ FET—Fisher exact test was performed in case one of the cells contained less than 5 observations.

**Table 5:** Differences in frequency of stroke type between male and female among each ethnic group.

Minority Communities	Sex	Stroke Type										*p*-Value from Chi^2^ Test
		Large Vessel		Cardio-Embolic		Small Vessel		TIA\Uncertain		Intracerebral Hemorrhage		
		N	%	N	%	N	%	N	%	N	%	
Druze (n = 232)	Male (n = 151)	15	9.90%	7	4.6% ^a^	84	55.60%	34	22.50%	7	4.60%	<0.05 *
	Female (n = 81)	8	9.90%	16	19.8% ^b^	35	43.20%	14	17.30%	6	7.40%	**(FET ^1^)**
Muslim (n = 81)	Male (n = 47)	5	10.40%	3	6.30%	26	54.20%	10	20.80%	1	2.10%	**0.89**
	Female (n = 34)	5	14.70%	2	5.90%	22	64.70%	2	5.90%	1	2.90%	
Bedouin (n = 40)	Male (n = 21)	3	14.30%	0	0%	14	66.70%	2	9.50%	1	4.80%	**0.96**
	Female (n = 19)	1	5.30%	1	5.30%	13	68.40%	2	10.50%	1	5.30%	
Christians (n = 20)	Male (n = 13)	3	23.10%	2	15.40%	7	53.80%	0	0% ^a^	1	7.70%	**0.93**
	Female (n = 7)	3	42.90%	0	0%	2	28.60%	2	28.6% ^b^	0	0	**(FET ^1^)**

* Significance *p* < 0.05. The letters indicate significantly higher (^b^), or lowest (^a^). ^1^ FET—Fisher exact test was performed in case one of the cells contained less than 5 observations. In comparing genders within each group, it was found that Druze women were more represented by cardio-embolic type of brain infarction (19.8%) compared with Druze men (4.6%) (*p* < 0.001).

## Data Availability

The raw dataset cannot be made publicly available due to patient confidentiality requirements. The data analyzed consists of medical records from patients at the participating hospitals where the study was conducted. Releasing identifiable patient data would violate regulations and institutional ethics approvals that safeguard protected health information and patient privacy rights.

## References

[1] Albright K.C., Huang L., Blackburn J., Howard G., Mullen M., Bittner V., Muntner P., Howard V. (2018). Racial differences in recurrent ischemic stroke risk and recurrent stroke case fatality. Neurology.

[2] Yang Q., Tong X., Schieb L., Vaughan A., Gillespie C., Wiltz J.L., King S.C., Odom E., Merritt R., Hong Y. (2017). Vital signs: Recent trends in stroke death rates—United States, 2000–2015. MMWR Morb. Mortal Wkly. Rep..

[3] Rinaldo L., Rabinstein A.A., Cloft H., Knudsen J.M., Castilla L.R., Brinjikji W. (2019). Racial and ethnic disparities in the utilization of thrombectomy for acute stroke: Analysis of data from 2016 to 2018. Stroke.

[4] Boot E., Ekker M.S., Putaala J., Kittner S., De Leeuw F.-E., Tuladhar A.M. (2020). Ischaemic stroke in young adults: A global perspective. J. Neurol. Neurosurg. Psychiatry.

[5] Wang Y., Rudd A.G., Wolfe C.D.A. (2013). Age and ethnic disparities in incidence of stroke over time: The South London stroke register. Stroke.

[6] Dehlendorff C., Andersen K.K., Olsen T.S. (2015). Sex Disparities in Stroke: Women Have More Severe Strokes but Better Survival Than Men. J. Am. Heart Assoc..

[7] Madsen T.E., Howard G., Kleindorfer D.O., Furie K.L., Oparil S., Manson J.E., Liu S., Howard V.J. (2019). Sex Differences in Hypertension and Stroke Risk in the REGARDS Study: A Longitudinal Cohort Study. Hypertension.

[8] Uchida K., Yoshimura S., Sakai N., Yamagami H., Morimoto T. (2019). Sex Differences in Management and Outcomes of Acute Ischemic Stroke with Large Vessel Occlusion. Stroke.

[9] Kamin Mukaz D., Zakai N.A., Cruz-Flores S., McCullough L.D., Cushman M. (2020). Identifying Genetic and Biological Determinants of Race-Ethnic Disparities in Stroke in the United States. Stroke.

[10] van der Weerd N., van Os H.J., Ali M., Schoones J.W., van den Maagdenberg A.M., Kruyt N.D., Siegerink B., Wermer M.J. (2021). Sex Differences in Hemostatic Factors in Patients with Ischemic Stroke and the Relation with Migraine—A Systematic Review. Front. Cell Neurosci..

[11] Manwani B., Fall P., Zhu L., O’reilly M.R., Conway S., Staff I., McCullough L.D. (2021). Increased P450 aromatase levels in post-menopausal women after acute ischemic stroke. Biol. Sex Differ..

[12] Elkind M.S.V., Lisabeth L., Howard V.J., Kleindorfer D., Howard G. (2020). Approaches to studying determinants of racial-ethnic disparities in stroke and its sequelae. Stroke.

[13] Greenberg E., Treger I., Schwarz J. (2011). Age, gender and risk factor disparities in first-stroke jewish and arab patients in Israel undergoing rehabilitation. Israel Med. Assoc. J..

[14] Koton S., Geva D., Streifler J.Y., Harnof S., Pougach Y., Azrilin O., Hadar S., Bornstein N.M., Tanne D. (2018). Declining rate and severity of hospitalized stroke from 2004 to 2013: The National Acute Stroke Israeli Registry. Stroke.

[15] Simaan N., Filioglo A., Cohen J.E., Lorberboum Y., Leker R.R., Honig A. (2022). Effects in Israel of Arab and Jewish Ethnicity on Intracerebral Hemorrhage. J. Clin. Med..

[16] Saad J., Ryder C.H., Hasan M., Keigler G., Badarny S. (2023). Primary Intracranial Hemorrhage: Characteristics, Distribution, Risk Factors, and Outcomes—A Comparative Study between Jewish and Arab Ethnic Groups in. J. Clin. Med..

[17] Telman G., Kouperberg E., Sprecher E., Yarnitsky D. (2010). Ethnic differences in ischemic stroke of working age in northern Israel. J. Stroke Cerebrovasc. Dis..

[18] Melnikov S., Itzhaki M., Koton S. (2018). Age-group and gender differences in stroke knowledge in an Israeli Jewish adult population. J. Cardiovasc. Nurs..

[19] Zimhony N., Abu-Salameh I., Sagy I., Dizitzer Y., Oxman L., Yitshak-Sade M., Novack V., Horev A., Ifergane G. (2018). Increase in ischemic stroke incident hospitalizations among Bedouin Arabs during Ramadan month. J. Am. Heart Assoc..

[20] Itzhaki M., Koton S. (2014). Knowledge, perceptions and thoughts of stroke among Arab-Muslim Israelis. Eur. J. Cardiovasc. Nurs..

[21] Awawdi K., Armon C., Kimiagar I., Tarabeih M., Rakia R.A. (2021). Post-Stroke Quality of Life Outcomes After Instituting New Stroke Care Quality Indicators. Eur. J. Med. Health Sci..

[22] Kamal H., Khodery M., Elnady H., Borai A., Schaefer J.H., Fawi G., Steinmetz H., Foerch C., Spitzer D. (2021). Adherence to Antithrombotic Treatment and Ischemic Stroke Recurrence in Egypt and Germany: A Comparative Analysis. Cerebrovasc. Dis..

[23] Paciaroni M., Ince B., Hu B., Jeng J.-S., Kutluk K., Liu L., Lou M., Parfenov V., Wong K.S.L., Zamani B. (2019). Benefits and Risks of Clopidogrel vs. Aspirin Monotherapy after Recent Ischemic Stroke: A Systematic Review and Meta-Analysis. Cardiovasc. Ther..

[24] Ben-Arye E., Shapira C., Keshet Y., Hogerat I., Karkabi K. (2009). Attitudes of Arab-Muslims toward integration of complementary medicine in primary-care clinics in Israel: The Bedouin mystery. Ethn. Health.

[25] Pharr J.R., Moonie S., Bungum T.J., Al-Faris E.A., Allam M.F., Bendtsen P. (2012). The impact of unemployment on mental and physical health, access to health care and health risk behaviors. ISRN Public Health.

[26] Krug G., Eberl A. (2018). What explains the negative effect of unemployment on health? An analysis accounting for reverse causality. Res. Soc. Strat. Mobil..

[27] Habibi-Koolaee M., Shahmoradi L., Niakan Kalhori S.R., Ghannadan H., Younesi E. (2018). Prevalence of Stroke Risk Factors and Their Distribution Based on Stroke Subtypes in Gorgan: A Retrospective Hospital-Based Study—2015–2016. Neurol. Res. Int..

[28] Kolominsky-Rabas P.L., Weber M., Gefeller O., Neundoerfer B., Heuschmann P.U. (2001). Epidemiology of Ischemic Stroke Subtypes According to TOAST Criteria. Stroke.

[29] Bekiaridou A., Samaras A., Kartas A., Papazoglou A.S., Moysidis D.V., Patsiou V., Zafeiropoulos S., Ziakas A., Giannakoulas G., Tzikas A. (2022). Sex-Related Differences in Clinical Outcomes in Patients with Atrial Fibrillation and Coronary Artery Disease: A Sub-Study of the MISOAC-AF Randomized Controlled Trial. J. Clin. Med..

[30] Evers-Dörpfeld S., Aeschbacher S., Hennings E., Eken C., Coslovsky M., Rodondi N., Beer J.H., Moschovitis G., Ammann P., Kobza R. (2022). Sex-specific differences in adverse outcome events among patients with atrial fibrillation. Heart.

[31] Manelis G., Shasha S. (1973). Atherosclerotic Heart Disease among the Druzes in Israel. Singap. Med. J..

[32] Yan H., Liu B., Meng G., Shang B., Jie Q., Wei Y., Liu X. (2017). The influence of individual socioeconomic status on the clinical outcomes in ischemic stroke patients with different neighborhood status in Shanghai, China. Int. J. Med. Sci..

[33] Haddad-Haj Yahya N., Assaf R. (2017). The Arab Society in Israel, Socio-Economic Status and Future Outlook.

